# Engineering tomato disease resistance by manipulating susceptibility genes

**DOI:** 10.3389/fgeed.2025.1537148

**Published:** 2025-02-10

**Authors:** Duoduo Wang, Palash Mandal, Md Sazan Rahman, Lirong Yang

**Affiliations:** ^1^ Department of Agriculture, Nutrition, and Food Systems, University of New Hampshire, Durham, NH, United States; ^2^ School of Pharmacy and Pharmaceutical Science, Cardiff University, Cardiff, United Kingdom

**Keywords:** CRISPR, crop improvement, immunity, susceptibility gene, tomato

## Abstract

Various pathogens severely threaten tomato yield and quality. Advances in understanding plant-pathogen interactions have revealed the intricate roles of resistance (R) and susceptibility (S) genes in determining plant immunity. While R genes provide targeted pathogen resistance, they are often vulnerable to pathogen evolution. Conversely, S genes offer a promising avenue for developing broad-spectrum and durable resistance through targeted gene editing. Recent breakthroughs in CRISPR/Cas-based technologies have revolutionized the manipulation of plant genomes, enabling precise modification of S genes to enhance disease resistance in tomato without compromising growth or quality. However, the utilization of the full potential of this technique is challenging due to the complex plant-pathogen interactions and current technological limitations. This review highlights key advances in using gene editing tools to dissect and engineer tomato S genes for improved immunity. We discuss how S genes influence pathogen entry, immune suppression, and nutrient acquisition, and how their targeted editing has conferred resistance to bacterial, fungal, and viral pathogens. Furthermore, we address the challenges associated with growth-defense trade-offs and propose strategies, such as hormonal pathway modulation and precise regulatory edits, to overcome these limitations. This review underscores the potential of CRISPR-based approaches to transform tomato breeding, paving the way for sustainable production of disease-resistant cultivars amidst escalating global food security challenges.

## Introduction

Tomato (*Solanum lycopersicum* L.) is one of the most economically important horticulture crops, representing one of the top produced and consumed vegetables worldwide. Tomatoes are popular as both fresh and processed vegetable that benefit human health with bioactive compounds like flavonoids, lycopene, and ascorbic acid (Borguini and Ferraz Da Silva Torres, 2009). In the context of rapid climate change, adverse environmental factors limit tomato growth and result in substantial yield loss and fruit quality deterioration (Bacelar et al., 2024). Along with these limiting factors, infectious diseases caused by a wide range of pathogens pose significant threats to crop yield and quality (Potnis et al., 2015; Zhang et al., 2022).

Co-evolution of plants and their surrounding pathogenic microorganisms has enabled plants to evolve a two-tiered immune system to combat pathogen infections (Jones and Dangl, 2006). The first line of plant immunity, known as pattern-triggered immunity (PTI), offers broad-spectrum defense response, which is triggered when plant cell surface receptors sense pathogen-associated molecular patterns (PAMPs) or damage-associated molecular patterns (DAMPs). Many pathogens can secrete virulent effectors into host cells, facilitating infection by breaching the first line of defense. Thus, plants employed a second layer of defense, known as effector-triggered immunity (ETI) to counter pathogens. The ETI is a more specialized and robust defense that is triggered when plant intracellular immune receptors, mostly the nucleotide-binding/leucine-rich-repeat receptors (NB-LRRs or NLRs) class, detect and recognize pathogen effector proteins, often leading to hypersensitive response (HR), localized cell death and induction of systemic acquired resistance (Jones et al., 2024). PTI and ETI act synergistically and mutually potentiate each other to trigger a robust defense against pathogen (Ngou et al., 2021; Yuan et al., 2021). Numerous transcriptional factors and hormones highly regulate the interplay between PTI and ETI.

The competition of resistance (R) genes and susceptibility (S) genes during plant-pathogen interactions determines if the plant will resist or be affected by a disease during interactions with pathogens. R genes are broadly grouped into two classes: typical R genes, which include NLRs and membrane-localized receptor-like kinases or proteins (RLKs/RLPs); and atypical R genes, which possess diverse architectures and functions associated with transcriptional regulation, kinases, translocation of substrates and hormone signaling (Sun et al., 2024). Typical R genes in plants mainly recognize specific pathogenic proteins or effectors. Gaining disease resistance by incorporating typical R genes can be challenging because these genes constantly evolve under strong positive selection due to the rapid evolution of pathogenic effectors. In contrast, many atypical R genes exhibit broad-spectrum and durable resistance and thus can be promising candidates for the resistant crop breeding (Sun et al., 2024).

S genes are defined as any plant genes that allow compatibility with pathogens and facilitate infection, and they can be further categorized into three subclasses depending on diverse functions (van Schie and Takken, 2014). The first type of S genes helps pathogens enter the host plant by regulating cell wall structure, cuticle properties, and stomata opening. The second type suppresses the immune response of plants, especially through transcriptional and hormonal regulation. The third type allows pathogens to access nutrients and grow by controlling sugar transport and metabolite production (van Schie and Takken, 2014; Koseoglou et al., 2022).

Pesticides have been used worldwide to control plant disease for over half a century. However, their overuse presents a substantial risk to the environment, ecological stability, and human health (Kimm et al., 2017). Breeding disease-resistant crops using new breeding technologies including gene editing is an effective and sustainable strategy for plant protection (Manzoor et al., 2024). Natural genetic variation and artificially induced genetic diversity, combined with modern molecular and genetic tools, including deep sequencing technologies and gene editing, have accelerated the discovery of genomic regions or alleles associated with immunity in tomato. So far, dozens of genes related to plant immunity, including R genes and S genes in tomato have been mapped and cloned (Rothan et al., 2019), and the role of these genes in regulating disease resistance was characterized in CRISPR edited tomato plants (Table 1).

**TABLE 1:** Application of CRISPR/Cas-based gene editing tool for engineering resistance against pathogens in tomato plants.

Target gene	Function	Target pathogen/disease	Tomato cultivar	Method	Outcome	Refs.
*SlJAZ2*	Co-receptor of coronatine in stomatal guard cells	*Pseudomonas syringae* pv. tomato (*Pto*) DC3000; *Botrytis cinerea (B. cinerea)*	Moneymaker	CRISPR/Cas9; Agrobacterium -mediated stable transformation	Resistance to *Pto* DC3000; Unaltered resistance to *B. cinerea*	Ortigosa et al. (2019)
*eIF4E1*	Aids the initiation of protein translation	Pepper mottle virus (PepMoV); Tobacco, etch virus (TEV)	Micro-Tom	CRISPR/Cas9; Agrobacterium -mediated stable transformation	Enhanced resistance to PepMoV without effect on growth; Unchanged resistance to TEV	Yoon et al. (2020)
*SlPMR4*	Callose synthesis at infection sites; repress salicylic acid (SA)-associated defense pathway	Powdery mildew (PM)	Moneymaker	CRISPR/Cas9; Agrobacterium -mediated stable transformation	Increased resistance to PM	Santillán Martínez et al. (2020)
*SlPMR* *4*	Callose synthesis at infection sites; repress SA-associated defense pathway	*Phytophthora infestans* (*P. infestans*)	San Marzano (SM) and Oxheart (OX)	CRISPR/Cas9; Agrobacterium -mediated stable transformation	Reduced susceptibility *to P. infestans*	Li et al. (2022)
*LeEIX1*	Cell-surface receptor binding to fungal elicitor EIX; attenuating EIX induced immune response	*Trichoderma harzianum* (*T. harzianum*)	M82	CRISPR/Cas9; Agrobacterium -mediated stable transformation	Increased response to *T. harzianum*	Leibman-Markus et al. (2021)
*SlWak1*	Flagellin-mediated pattern triggered immunity (PTI)	*Pseudomonas*. *syringae pv.* Tomato DC3000	Rio-Grande PtoR (RG-PtoR)	CRISPR/Cas9; Agrobacterium -mediated stable transformation	Compromised in PTI induced by flagellin	a Zhang et al. (2020a)
*SlPelo*	Messenger RNA surveillance factor involved in ribosome recycling	Tomato yellow leaf curl virus (TYLCV)	BN-86	CRISPR/Cas9; Agrobacterium -mediated stable transformation	TYLCV resistance by restricting the viral proliferation	Pramanik et al. (2021)
*SlMlo1*	Susceptibility factor of fungal PM	PM	BN-86	CRISPR/Cas9; Agrobacterium -mediated stable transformation	Complete resistance to PM fungus	Pramanik et al. (2021)
*SlPLC2*	Susceptibility gene facilitating pathogen infection and proliferation	*B. cinerea*	MM-Cf0 cultivar	CRISPR/Cas9; Agrobacterium -mediated stable transformation	Enhanced resistance to *B. cinerea*	Perk et al. (2023)
*SlBBX20*	Suppressing Jasmonic acid (JA) signaling	*B. cinerea*	Alisa Craig’	CRISPR/Cas9; Agrobacterium -mediated stable transformation	Enhanced resistance to *B. cinerea*	Luo et al. (2023)
*SRFR1*	Adaptor protein negatively regulating effector triggered immunity (ETI)-associated transcriptional immune responses	*Pto* DC3000; *Fusarium oxysporum* f. sp. *lycopersici* (*FOL*); *B. cinerea*	M82	CRISPR/Cas9; Agrobacterium -mediated stable transformation	Enhance resistance to *Pto* DC3000; Enhanced susceptibility to *FOL* and *B. cinerea*	Son et al. (2021)
*SlHyPRP1*	Hybrid proline-rich protein involved in cell-wall signaling, plant development, and stress responses	*Pto* DC3000; *FOL*	Hongkwang and 15T01 local tomato varieties	CRISPR/Cas9; Agrobacterium -mediated stable transformation	Enhanced resistance to *Pto* DC3000; Enhanced susceptibility to *FOL*	Tran et al. (2023)
*SlNRX1*	Nucleoredoxin modulating the redox states of immune signaling proteins and negatively regulating plant immunity	*Pseudomonas yringae pv. maculicola* (Psm) ES4326	Micro-Tom	CRISPR/Cas9; Agrobacterium -mediated stable transformation	Enhanced resistance to *Psm* ES4326	Cha et al. (2023)
*I2*	Nucleotide-binding (NB) leucine-rich repeat (LRR) (or NLR) protein	*P. infestans*;*FOL*	*Nicotiana . benthamiana*	Agroinfiltration	Expanded resistance profile to *P. infestans* and *FOL*	Giannakopoulou et al. (2015)
*PL*	Pectate lyase involved in cell wall degradation during fruit ripening	*B. cinerea*	Alisa Craig	CRISPR/Cas9; Agrobacterium -mediated stable transformation	Reduced susceptibility to *B. cinerea*	Silva et al. (2021)
*SlDMR6-1*	SA-5 hydroxylase involved in the SA catabolic pathway	*Pto* DC3000*. Xanthomonas. g*ardneri*;* *Phytophthora capsici;; Xanthomonas perforans;* *Pseudoidium neolycopersici*	Fla. 8000	CRISPR/Cas9; Agrobacterium -mediated stable transformation	Enhanced resistance with no evident growth penalty	Thomazella et al. (2021)

Disease resistance can be ensured via the incorporation and introgression of desired R genes into crops through different breeding approaches, including conventional breeding and transgenic technology (Derbyshire et al., 2024). However, R gene associated resistance is mostly disrupted because their targeted effectors are generally under strong negative selection (Sacristán et al., 2021; Bent and Mackey, 2007). Additionally, the incorporation of R genes into host genome via conventional breeding can be time-consuming or results in genetically modified organisms (GMOs) by means of genetic engineering techniques, which will provoke public concern (Rozas et al., 2022). A better strategy is using mutated S genes for sustainable and broad-spectrum resistance. Various gene-editing technologies, particularly CRISPR/Cas-based gene editing tools, have opened up new avenues to precisely engineer S genes for resistance breeding. CRISPR/Cas9 and its derivatives have been developed for a wide variety of applications, including gene knockout, gene knock-in, gene regulation, and epigenetic editing (Altpeter et al., 2016; Čermák et al., 2015; Tyumentseva et al., 2023). Recently, gene editing has greatly expedited our understanding of plant-pathogen interaction and the development of host resistance against various biotic stresses in many crops (Zhao et al., 2022). This mini-view summarizes the recent applications of genome editing technology in developing disease-resistant tomato cultivars, discusses the current obstacles that may restrict the use of CRISPR in breeding disease-resistant crops, and proposes some viewpoints that may help overcome these challenges.

## Gene editing of S genes in tomato for improved host immunity

Pathogens usually need to break through the plant cell wall to infect a plant. This process activates various cell wall structure-related genes. Recent studies showed that knocking out a cell wall structure-related gene *pectate lyase* (*PL*) in tomato cultivar Ailsa Craig (AC) using CRISPR/Cas9 resulted in significantly reduced degradation of pectin, increased fruit firmness and resistance against fungal disease (Wang et al., 2018; 2019; Silva et al., 2021; Ortega-Salazar et al., 2024), suggesting a strong link between pectin and cell wall-mediated plant immunity.

Instead of breaching the cell wall, some pathogens enter the host apoplast via entry portals like stomata with the help of S genes. *SlJAZ2* which encodes a major co-receptor of coronatine (COR) in tomato stomatal guard cells could facilitate *Pseudomonas syringae pv. tomato* (*Pto*) DC3000 colonization by stimulating stomata opening. Knocking out *SlJAZ2* in the tomato cultivar Moneymaker using CRISPR/Cas9 resulted in enhanced resistance to (*Pto*) DC3000 while did not affect resistance to the necrotrophic fungal pathogen *Botrytis cinerea* (*B. cinerea*) that does not rely on stomata for penetration (Ortigosa et al., 2019). The tomato S gene *Phospholipase C2* (*SlPLC2*), which can be induced by fungal elicitor xylanase, was required for *B. cinerea* proliferation. Knock-downing the expression of *SlPLC2* by virus-induced gene silencing and knocking out *SlPLC2* by CRISPR/Cas9 in the tomato cultivar Moneymaker without Cf resistance genes (MM-Cf0) resulted in enhanced resistance to *B. cinerea,* accompanied by decreased reactive oxygen species (ROS) production and altered salicylic acid (SA) and jasmonic acid (JA) signaling pathways (Gonorazky et al., 2016; Perk et al., 2023). Cell surface localized receptors can bind microbial elicitors and mediate plant defense response. In tomato plants, cell-surface decoy receptor LeEIX1 has the ability to bind ethylene-inducing xylanase (EIX), a fungal elicitor secreted by *Trichoderma* spp, and attenuate EIX-induced signaling and host defense (Ron and Avni, 2004). Knocking out *LeEIX1* in tomato M82 cultivar using CRISPR/Cas9 led to stronger host immune activation and enhanced disease resistance against *Trichoderma* in *LeEIX1*-edited lines compared with wild-type (WT) control plants (Leibman-Markus et al., 2021).

Some S genes facilitate pathogen survival by providing nutrients or supporting microbial metabolism. One of the best characterized S gene family is *mildew resistance locus o* (*Mlo*) which encodes membrane-associated proteins and was reported to confer susceptibility to powdery mildew (PM) disease in many plant species (Acevedo-Garcia et al., 2014). Targeted mutagenesis of *SlMlo1*, the major contributor to PM susceptibility, in both tomato BN-86 and Moneymaker cultivars using CRISPR/Cas9 resulted in fully resistant plants to the PM fungus without compromising plant growth and fruit development (Nekrasov et al., 2017; Pramanik et al., 2021). *SlPelo* was previously discovered to encode a messenger RNA surveillance factor and render susceptibility to *Tomato yellow leaf curl virus* (TYLCV) (Lapidot et al., 2015). More recently, the role of *SlPelo* in regulating TYLCV infection in the elite tomato cultivar BN-86was validated in *SlPelo*-edited mutants generated by CRISPR/Cas9, and the results indicated that *SlPelo* was a susceptibility factor of yellow leaf curl disease caused by TYLCV (Pramanik et al., 2021). Another well-studied S gene related to PM is *POWDERY MILDEW RESISTANT 4* (*PMR4*). *PMR4* was reported to inhibit SA defense signaling pathway (Nishimura et al., 2003). CRISPR/Cas-9 mediated mutagenesis of *SlPMR4* in susceptible tomato cultivar moneymaker resulted in reduced susceptibility to PM, accompanied by a higher occurrence of hypersensitive response-like cell death at infection sites in the CRISPR edited line compared with a control plant, which was likely to be induced by SA signaling pathway (Santillán Martínez et al., 2020). More recently, knocking out *SlPMR4* in two widely grown Italian tomato cultivars including San Marzano (SM) and Oxheart (OX) by CRISPR/Cas9, indicated that *SlPMR4* conferred susceptibility to Late Blight (LB), a fungal disease caused by *Phytophthora infestans* (Li et al., 2022). Genes required for viruses to maintain their lifecycle can also be regarded as S genes. Yoon et al. (2020) generated CRISPR/Cas9-derived mutations in the *eukaryotic translation initiation factor 4E* (*eIF4E*) in the tomato cultivar Micro-Tom, and evaluated the role of *eIF4E* in Potyvirus resistance. Results demonstrated that *eIF4E* was a susceptible factor which is necessary for pepper mottle virus (PepMoV) infection (Yoon et al., 2020).

The third class of S genes act as negative regulators of host innate immune response, many of which regulate hormone signaling pathways. Disabling such S genes is likely to be a promising strategy to obtain durable and broad-spectrum disease resistance. Inactivating a tomato *DOWNY MILDEW RESISTANCE* gene (*SlDMR6-1*) in the tomato Fla. 8000 variety using CRISPR/Cas9 enhanced resistance to bacterial, oomycete, and fungal pathogens, correlating with increased SA (Thomazella et al., 2021). Moreover, SlDMR6-1 displayed SA-5 hydroxylase activity, which could explain the increased SA level in the *SlDMR6-1* homozygous mutants (Thomazella et al., 2021). More recently, CRISPR/Cas9 was employed to edit two tomato *NUCLEOREDOXIN* (*SlNRX*) genes including *SlNRX1* and *SlNRX2* in the Mocro-Tom cultivar, and characterization of the CRISPR edited plants unraveled the negative role of *SlNRX1,* a member of the nucleocytoplasmic THIOREDOXIN subfamily, in modulating SA-dependent immune response to both bacterial and fungal infection in tomato (Cha et al., 2023).

Another important plant hormone JA regulates various biological processes, including plant immunity, through complex modules involving multiple transcription factors (TFs) or regulators. Manipulating a number of *MYC2-TARGETED BHLH* (*MTB*) genes which negatively regulate JA-mediated defense response in the tomato AC cultivar by CRISPR/Cas9 resulted in stronger resistance to herbivore attack compared to WT plants, without compromising normal plant growth (Liu et al., 2019). By integrating CRISPR/Cas9 with RNA sequencing, *SlBBX20*, a gene belonging to the B-box (BBX) family in tomato, was knocked out in the tomato AC cultivar and found to negatively regulate resistance to *B. cinerea* in tomato plants by attenuating JA signaling (Luo et al., 2023).

The contrasting effect of host genes in regulating resistance to different pathogens was observed in many plants. A functional study of tomato *SRFR1* using CRISPR-based gene editing tool revealed its negative role in immune response to *Pto* DC3000 via regulating SA-pathway defense genes, while it functioned as a positive regulator to necrotrophic pathogens *Fusarium oxysporum f. sp. lycopersici* (*FOL*) by modulating JA/ethylene genes (Son et al., 2021). Similarly, a tomato gene *SlHyPRP1*encoding a proline-rich protein involved in cell wall signaling was reported as a negative regulator of defense to *Pto* DC3000 but a positive regulator of immunity to *FOL* (Tran et al., 2023). It has been suggested that defense against biotrophic pathogens is largely regulated by SA signaling pathway while JA/ethylene mainly dominates in facilitating host defense response against necrotrophic pathogens (Glazebrook, 2005). In addition to JA and SA, other hormones including auxins, cytokinins (CK), abscisic acid, gibberellins (GA), brassinosteroids, strigolactones, as well as nitric oxide, are likely to act antagonistically in the regulation of plant-pathogen interactions (Huang et al., 2020). The regulation of plant defense is largely dependent on multiple hormone pathways often interconnected by complex transcription module. The antagonistic role of host genes in regulating defense against different types of pathogens might be explained by different attack strategies of pathogens and complex strategies of host plants to counteract the invasion of pathogens.

Gene editing technology has been used to elucidate the molecular mechanisms of R genes in regulating immune response in tomato. For instance, the role of a cell wall-associated kinase gene named *SlWak1* was characterized in CRISPR mutant lines generated in the Grande PtoR (RG-PtoR) genetic background, suggesting SlWak1, acting in a complex with Fls2 and Fls3, positively regulate immune signaling at later stages of PTI in the apoplast upon the inoculation of *P. syringae* pv. tomato (*Pst*) (Zhang et al., 2020a). So far, more than 150 genes in tomato associated with host immunity to various pathogens have been edited by gene editing technologies, and detailed information can be found in Plant Genome Editing Database (PGED) (Zheng et al., 2019). Among them, 63 candidate genes were further molecularly characterized in CRISPR edited lines for their gRNA efficiency, specificity of modifications and heritability of the mutations (Zhang et al., 2020b). Many of the selected genes encode cell surface localized pattern recognition receptors (PRRs), kinases, transporters and TFs, and their role in plant immunity requires further investigation.

## Discussion

Incorporating R genes from natural germplasm resources into cultivated species by classical or transgenic breeding has been used for enhancing disease resistance in many crops including tomato. However, this is a lengthy and laborious process. R genes usually defend against specific pathogens, and their effectiveness may not last long because they often fail to recognize frequently mutated pathogen effectors of newly evolved pathogens. Engineering R genes using CRISPR-based technology to achieve broad-spectrum resistance might be a promising solution to this challenge. Giannakopoulou et al. (2015) engineered a tomato *NLR* gene named *I2* and investigated if the mutation in *I2* could alter plant response to the pathogen effector AVR3a in *Nicotiana benthamiana* by agroinfiltration assay. It was shown that mutation of *I2* resulted in markedly increased response to AVR3a, suggesting altered resistance to pathogens could be achieved by engineering synthetic immune receptors (Giannakopoulou et al., 2015).

**FIGURE 1:**
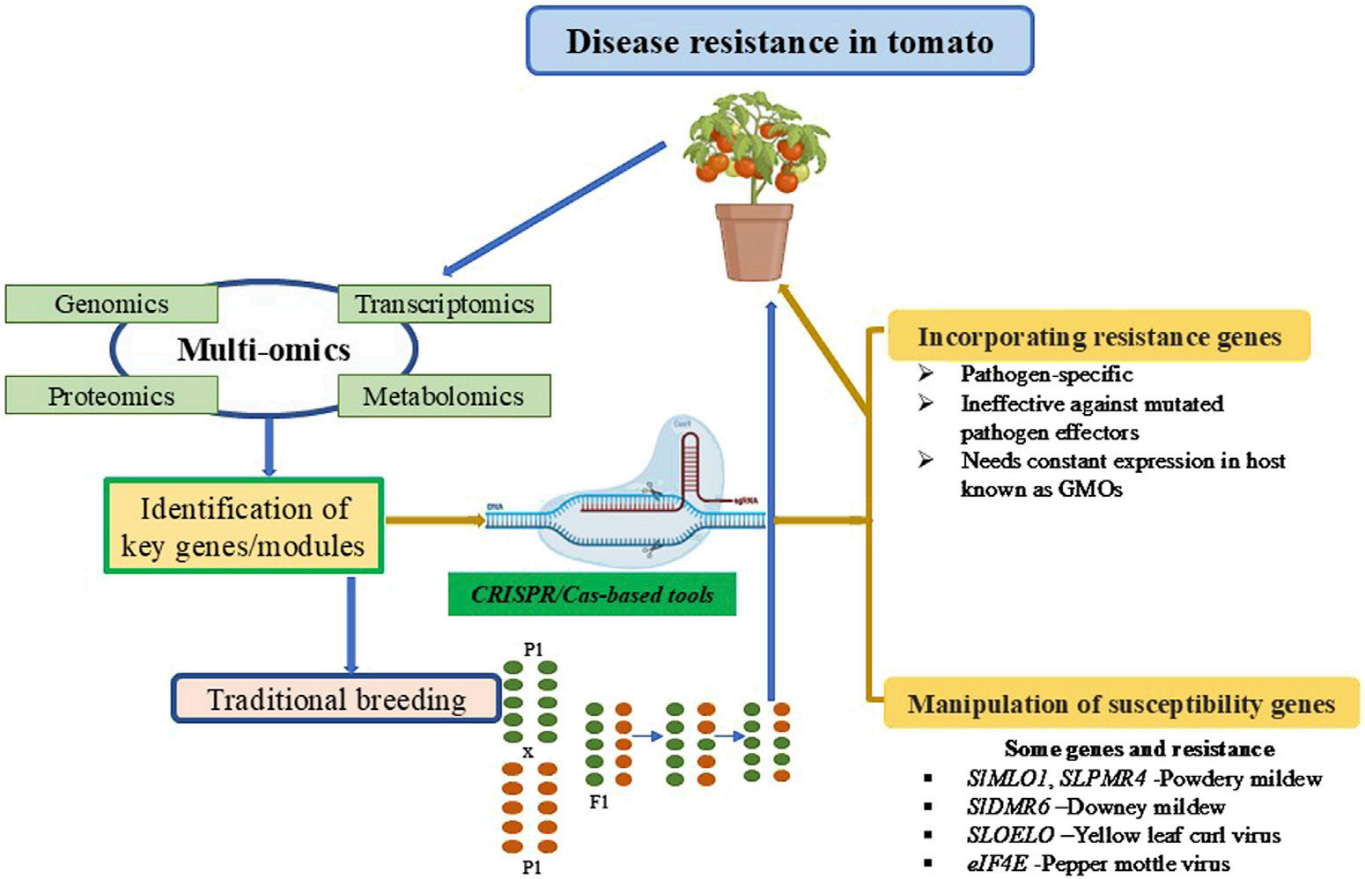
An integrated framework for improving disease resistance in tomatoes through multi-omics approaches and genome editing technologies. Genomics, transcriptomics, proteomics, and metabolomics are utilized to identify key genes or modules associated with disease resistance. These insights are applied through two complementary strategies: traditional breeding methods and CRISPR/Cas-based genome editing tools. CRISPR/Cas technology facilitates the incorporation of resistance (R) genes and the manipulation of susceptibility (S) genes, enabling precise genetic modifications to enhance tomato disease resistance. This systematic approach combines modern molecular tools with conventional practices to develop robust, disease-resistant tomato cultivars.

Another approach to achieve resistance is manipulating S genes, a class of plant genes that facilitate pathogen penetration and proliferation or supports compatibility with pathogens. Enhanced disease resistance can be achieved by manipulating S genes using gene editing. Several strategies have been developed to generate transgene-free CRISPR edited crops which are defined as non- GMO; these include elimination of transgenic sequences via genetic segregation, and transient expression of CRISPR/Cas9 editor via ribonucleoprotein (RNP)-mediated CRISPR genome editing method (Gu et al., 2021; Ahmad et al., 2023). The options for utilizing both R and S genes to develop disease-resistant genotypes are summarized in Figure 1. However, the defense-growth trade-off or fitness cost of silencing S genes is common in plants, which are species and condition-specific. Modifying the regulatory element precisely might be a solution. Plant growth-defense trade-off has remained a challenge when engineering disease resistance. Numerous studies have suggested enhanced defense usually results in inhibited growth and development, and this process is modulated by complex network involving interactions between multiple regulators and hormones (Giolai and Laine, 2024; He et al., 2022). In tomato, several modules were reported to fine-turn trade-off of plant growth-defense. For example, using a systematical approach including CRISPR/Cas9 and transcriptional regulation methods, the RALF2-FER-MYB63 module was found to fine-tune root growth and resistance against *FOL* through regulating the deposition of lignin in tomato cultivar Condine Red (Fan et al., 2024). JA was shown to involve in maintaining a balance between lateral root (LR) development and root-knot nematode (RKN) susceptibility via SlMYB-mediated transcriptional regulation in tomato (Zhao et al., 2023). A more recent study using three different immunity elicitors to investigate the effect of systemic acquired resistance (SAR) and induced systemic resistance (ISR) pathways on tomato development indicated that growth and defense could be positively correlated through alterations to the CK/GA balance (Leibman-Markus et al., 2023). This study challenges the classic model of the growth-defense trade-off, suggesting that growth promotion and induced resistance can be co-dependent. It further shows that defense priming can occur within a specific developmental window and that growth-defense trade-off can be uncoupled through the modulation of certain hormonal pathways. Therefore, uncoupling the antagonism between hormonal pathways opens new avenues for applying gene editing tools to develop crops with enhanced biotic stress resistance. So far, only a limited number of S genes have been identified and manipulated in tomato for conferring resistance to pathogens. A wealth of available tomato genetic resources and multi-omics dataset, along with newly developed web-based platforms, including the Tomato multi-omics data Analysis Platform (TomAP) (http://bioinformatics.cau.edu.cn/TomAP/) (Cao et al., 2024) and Solanaceae Information Resource (SoIR) (https://soir.bio2db.com) (Liu et al., 2024), will facilitate the identification of key genes or modules linked to immune response in tomato. Future research should also focus on developing novel CRISPR/Cas-based toolbox and using high-throughput genetic screens to improve the editing of disease resistance genes. The present review proposes that critical challenges in sustainable agriculture, such as dependency on pesticides and drawbacks of conventional methods, can be addressed by leveraging gene editing technology. Additionally, it proposes multi-omics approaches coupled with advanced gene editing tools provide an effective way for precisely engineering disease resistance for crop improvement.
